# The D-FISH Trial: A Randomized, Double-Blind, Non-Inferiority Trial Comparing Fish Processing By-Product-Derived Versus Synthetic Vitamin D_3_ Supplementation in Adults with Suboptimal 25-Hydroxyvitamin D

**DOI:** 10.3390/jcm15031186

**Published:** 2026-02-03

**Authors:** Federica Fogacci, Serra İlayda Yerlitaş Taştan, Cristina Scollo, Jessica Lago, Nicola Bertini, Gianni Sagratini, Arrigo F. G. Cicero, on behalf of the VITADWASTE Working Group

**Affiliations:** 1 Hypertension and Cardiovascular Risk Research Center, Medical and Surgical Sciences Department, Alma Mater Studiorum University of Bologna, 40100 Bologna, Italy; federica.fogacci@studio.unibo.it (F.F.); ilaydayerlitas340@gmail.com (S.İ.Y.T.); jessica.lago@studio.unibo.it (J.L.); nicola.bertini2@studio.unibo.it (N.B.); 2 Italian Society of Nutraceuticals (SINut), 40100 Bologna, Italy; gianni.sagratini@unicam.it; 3 Department of Medical Pharmacology, Medical Faculty, Ataturk University, Erzurum 25240, Turkey; 4 Department of Biostatistics, Erciyes University School of Medicine, Kayseri 38039, Turkey; 5 Cardiovascular Medicine Unit, IRCCS AOU BO, 40138 Bologna, Italy; cristina.scollo@aosp.bo.it; 6 Chemistry Interdisciplinary Project (ChIP), School of Pharmacy, University of Camerino, 62032 Camerino, Italy

**Keywords:** bioavailability, clinical trial, fish, protocol, supplementation, vitamin D

## Abstract

Vitamin D insufficiency remains common in adults, and supplementation with cholecalciferol (vitamin D_3_) is widely used to improve circulating 25-hydroxyvitamin D [25(OH)D]. At the same time, circular-economy strategies are increasingly applied to nutraceutical production, including the valorization of fish processing by-products as sources of lipid-soluble bioactives. However, clinical evidence directly comparing fish processing by-product-derived vitamin D_3_ with conventional synthetic vitamin D_3_ at commonly used nutritional doses remains limited. D-FISH is a single-center, randomized, double-blind, parallel-group, non-inferiority trial enrolling adults aged 18–60 years with a baseline plasma of 25(OH)D 20–40 ng/mL. Participants will be randomized (1:1) to receive either fish processing by-product-derived or synthetic vitamin D_3_ at the same nutritional dose (600 IU once daily with the evening meal) for 12 weeks. The primary endpoint is the between-group difference in the change in plasma 25(OH)D from day 0 to day 84, assessed against a pre-specified non-inferiority margin of 5 ng/mL, with analyses conducted in full analysis and per-protocol populations. Secondary endpoints include markers of mineral metabolism (calcium, phosphorus, PTH), fasting lipid profile, anthropometrics, and tolerability/safety outcomes; early 25(OH)D kinetics will be explored at 72 h, day 7, and day 28. The study will inform biochemical non-inferiority and short-term tolerability but is not powered to evaluate clinical outcomes (NCT07127796).

## 1. Introduction

Vitamin D is a fat-soluble vitamin with hormone-like properties that plays a central role in calcium and phosphorus homeostasis, primarily by enhancing intestinal mineral absorption and supporting bone mineralization [1,2]. Two main forms are recognized: vitamin D_2_ (ergocalciferol), mainly derived from plant sources, and vitamin D_3_ (cholecalciferol), which is synthesized in the skin upon exposure to ultraviolet B (UVB) radiation and is also present in animal-derived foods such as fatty fish, liver, and egg yolks [1,2]. In both clinical practice and research, circulating 25-hydroxyvitamin D [25(OH)D] is considered the most appropriate biomarker for assessing vitamin D status [2,3].

Beyond its established skeletal role, vitamin D has been investigated for broader biological effects, including potential contributions to muscle function and immune regulation [2,4]. This wider biological interest has also been translated into large-scale clinical research programs exploring possible links with non-skeletal outcomes, such as cancer, cardiovascular risk, respiratory effects, autoimmune diseases, diabetes, and mortality [4,5]. Evidence regarding lipid-related effects of vitamin D supplementation remains inconsistent, with umbrella evidence suggesting more reproducible (albeit modest) effects on triglycerides (TG), while findings across total, LDL-, and HDL-cholesterol fractions are mixed; therefore, cardiometabolic markers are included here as exploratory correlates rather than efficacy outcomes [6]. However, evidence from randomized controlled trials on extra-skeletal endpoints has often been heterogeneous or largely neutral, with methodological explanations including the enrollment of vitamin D-replete participants, reverse causality, and outcome-specific differences in trial design and baseline risk [4,5]. Accordingly, the clinical interpretation of vitamin D as a pleiotropic modulator remains an active area of debate, particularly regarding which baseline 25(OH)D levels should be considered optimal and for which outcomes [4,7].

At the regulatory level, the European Food Safety Authority (EFSA) has supported authorized health claims for vitamin D, including its contribution to normal absorption and utilization of calcium and phosphorus, maintenance of normal blood calcium concentrations, and maintenance of normal bones and muscle function, as well as normal immune function; EFSA has also substantiated a claim related to reduction in the risk of falling in older adults under defined conditions of use [4,8,9]. Notably, the operational definition of “suboptimal” vitamin D status varies across recommendations, reflecting different public health and clinical perspectives; an additional challenge is the incomplete standardization of 25(OH)D assays, which complicates comparisons across studies and limits data pooling and meta-analyses [4].

Despite these ongoing uncertainties, low 25(OH)D concentrations remain common across Europe and represent a relevant public health issue. In a large, standardized assessment of European populations, the prevalence of circulating 25(OH)D < 50 nmol/L (approximately <20 ng/mL) was estimated at around 40%, with higher rates during the extended winter period and markedly greater prevalence in dark-skinned ethnic subgroups [10,11]. Italian cohorts mirror this pattern, even in regions with substantial solar irradiation: in a representative adult cohort from southern Italy, more than half of participants had 25(OH)D concentrations < 20 ng/mL, while only a minority achieved levels considered adequate (≥30 ng/mL) [11,12]. Seasonal variability, limited time spent outdoors, ageing, and comorbidities are recognized contributors, indicating that low vitamin D status may persist even in ostensibly “sunny” settings [10,11,12]. In line with this concept, recent perspectives emphasize that endogenous synthesis alone may be insufficient to maintain recommended circulating 25(OH)D levels throughout the year, reinforcing the rationale for monitoring and seasonally targeted supplementation strategies [13]. Moreover, maintaining adequate vitamin D status over time has been proposed as a prerequisite for preserving putative protective autocrine/paracrine effects in several tissues, whereas late correction of deficiency might be less effective once disease processes are established [14].

From a nutritional standpoint, fish is often regarded as a key dietary source of vitamin D_3_; however, its actual contribution to population adequacy is constrained by substantial variability in vitamin D content across species, farming practices, and even within the same species. For example, farmed salmon has been reported to contain markedly lower levels of vitamin D than wild salmon, suggesting that food composition tables may be outdated or imprecise [15]. Studies from different markets confirm a wide range of vitamin D_3_ concentrations in both freshwater and marine fish, with some species—including lean fish such as tilapia—showing unexpectedly high values [16,17], as well as the measurable cholecalciferol content documented across commercially consumed fish species [18]. Importantly, even when fish provides meaningful vitamin D_3_ intake, dietary approaches alone are unlikely to eradicate deficiency at scale, given the gap between requirements and realistic consumption patterns [16]. Cooking practices may further influence vitamin D availability, although several domestic methods (including boiling, frying, and grilling) can retain substantial vitamin D content in selected species [17].

Accordingly, strategies to address vitamin D insufficiency include safe sunlight exposure, dietary interventions and food fortification, and supplementation. In routine practice, nutritional-dose vitamin D_3_ supplementation is frequently adopted for individuals with mild insufficiency or to maintain adequate levels, particularly during low-UVB months [11,19]. The EFSA Dietary Reference Values define an Adequate Intake (AI) for adults of 15 μg/day (600 IU/day) under conditions of minimal cutaneous vitamin D synthesis [20,21]. Oral administration is generally preferred in the general population, whereas parenteral routes are reserved for selected clinical settings such as malabsorption or vitamin D resistance [22,23]. Cholecalciferol is typically favored because of its safety profile and minimal monitoring requirements, while calcifediol may be considered in specific conditions, and calcitriol should be restricted to disorders characterized by impaired endogenous activation [22,23]. At nutritional doses, differences in delivery vehicle may be less relevant than the administered dose itself: in healthy adults supplemented with 10 μg/day vitamin D_3_ during late winter, fish oil capsules and solid multivitamin tablets produced similar increases in plasma 25(OH)D over four weeks [7]. This supports the premise that alternative vitamin D_3_ sources may be acceptable if they demonstrate comparable bioavailability and safety.

In parallel with clinical interest, sustainability and circular-economy frameworks are increasingly informing nutraceutical development. Fish and seafood processing generates substantial by-products that may serve as sources of lipid-soluble bioactives, including vitamin D_3_ [24,25]. Beyond extraction, ingredients derived from fish processing by-products are also being explored as functional platforms for vitamin delivery: calcined cuttlefish bone derived from shell by-products has demonstrated high loading efficiency for cholecalciferol and controlled-release kinetics in vitro, supporting the feasibility of side-stream-derived matrices for vitamin D_3_ delivery applications [26]. These approaches may reduce waste streams and reliance on conventional production routes; however, their translational value ultimately depends on demonstrating biological efficacy and safety comparable to established synthetic preparations when administered at commonly used nutritional doses [25]. To date, direct clinical comparisons between fish processing by-product-derived vitamin D_3_ and conventional synthetic vitamin D_3_ remain limited, and dedicated head-to-head trials are only now emerging.

The D-FISH trial was designed to address this gap by testing whether a fish processing by-product-derived vitamin D_3_ supplement is non-inferior to a synthetic vitamin D_3_ supplement in increasing plasma 25(OH)D concentrations over 12 weeks in healthy adults with suboptimal vitamin D status. This non-inferiority framework is particularly suited to sustainability-driven innovation, where demonstrating comparable efficacy and safety to an established standard may be sufficient to support adoption when additional environmental advantages are anticipated. In addition to the primary biochemical endpoint, the protocol includes markers of mineral metabolism and a structured assessment of tolerability and safety, while also exploring early 25(OH)D kinetics through intermediate sampling time points, thereby providing a granular characterization of response dynamics.

## 2. Methods

### 2.1. Study Design

D-FISH is a single-center, randomized, double-blind, parallel-group, non-inferiority trial designed to compare a fish processing by-product-derived vitamin D_3_ nutraceutical with a standard synthetic vitamin D_3_ supplement [27]. Participants will be randomly allocated in a 1:1 ratio to one of the two interventions and will receive study treatment for 12 weeks.

### 2.2. Study Setting

The study will be conducted at the outpatient clinics of the Cardiovascular Medicine Unit, IRCCS AOU di Bologna (Bologna, Italy), which will serve as the single recruiting and follow-up center. Blood samples will be collected on site and subsequently processed and analyzed at the Lipid Laboratory, Department of Medical and Surgical Sciences, Alma Mater Studiorum—University of Bologna.

### 2.3. Eligibility Assessment

Eligibility will be assessed at baseline based on medical history, concomitant therapies, and laboratory values. Full inclusion and exclusion criteria are summarized in Table 1.

Notably, clinically relevant malabsorption syndromes (where oral supplementation may be ineffective and parenteral strategies are often required) would be captured under the general exclusion criterion of any medical or surgical condition that would limit adherence to the study protocol. Participants will be advised to maintain their usual lifestyle habits throughout the study period, including habitual outdoor activities and sun exposure, and to avoid major intentional changes that could substantially modify vitamin D status.

### 2.4. Recruitment and Informed Consent

Potential participants will be identified among adults attending outpatient evaluations (e.g., for dyslipidemia or hypertension) and pre-screened for eligibility. Individuals meeting the inclusion criteria will receive written and verbal information about the study and will have the opportunity to ask questions before deciding whether to participate. Written informed consent will be obtained prior to any trial-specific procedures. Recruitment will span multiple months; the calendar period of enrolment will be recorded, and randomization is expected to distribute seasonal influences across groups. Seasonality-related variability will be considered in sensitivity analyses if imbalances are observed.

### 2.5. Study Products and Administration

Participants will receive one of two oral vitamin D_3_ formulations and will be instructed to take one capsule once daily with the evening meal for 12 weeks. This administration schedule was pre-specified in the approved protocol to standardize intake conditions across participants and to maximize the likelihood of administration in a postprandial setting, thereby limiting variability related to absorption. The intervention uses a nutritional low dose of vitamin D_3_ (600 IU/day), consistent with the EFSA Adequate Intake for adults under conditions of minimal cutaneous synthesis. Both study products are supplied as hard gelatin capsules and will be packaged and labelled in a manner designed to preserve blinding throughout the trial.

#### 2.5.1. Investigational Product: Fish Processing By-Product-Derived Vitamin D_3_

The investigational product contains fish-derived cholecalciferol (vitamin D_3_) obtained from sardine (*Sardina pilchardus*) processing by-products (whole fish), collected by CNR-IRBIM from a professional fishing fleet operating in the Adriatic Sea (Central Mediterranean Sea). Upstream handling follows a predefined cold-chain and timing workflow aimed at preserving the native lipid matrix. Specifically, the raw material is cut and homogenized and subsequently freeze-dried within a maximum of 5 days from catching. During the interval between capture and initiation of freeze-drying, the material is maintained at −20 °C, and after lyophilization, the resulting fish meal is stored at −20 °C until extraction.

The vitamin D_3_-containing fraction is obtained by supercritical CO_2_ extraction (SFE-CO_2_) performed at a supercritical fluids research pilot plant at ENEA. The resulting extract is stored at 4 °C and protected from light prior to formulation. Chemical characterization and standardization are conducted at the University of Camerino using HPLC-DAD on an Agilent 1260 Infinity II system coupled with a diode array detector. Vitamin D_3_ and 7-dehydrocholesterol (7-DHC) are quantified using a previously optimized method. In the extract intended for formulation, vitamin D_3_ and 7-DHC concentrations were 6.18 ± 0.54 μg/g and 789.77 ± 95.02 μg/g, respectively.

To further characterize the lipid matrix accompanying vitamin D_3_, fatty acids are derivatized to fatty acid methyl esters (FAMEs) and analyzed by GC–MS (Agilent 8890 gas chromatograph coupled to an Agilent 5977B mass spectrometer, Agilent Tehcnologies, Santa Clara, CA, USA). Compound identification is supported by the NIST 2020 library, and relative abundances are expressed as percentage peak areas. Overall, polyunsaturated fatty acids (PUFAs) represent the predominant fraction (>43%), followed by saturated fatty acids (~29%) and monounsaturated fatty acids (~19%). Docosahexaenoic acid (DHA) shows the highest relative abundance, and additional ω-3 PUFA species—including eicosapentaenoic acid (EPA) and docosapentaenoic acid (DPA)—are also detected.

For the final dosage form, the extract is microencapsulated at the University of Piemonte Orientale laboratories and formulated into hard gelatin capsules using a defined excipient system including PUFAs, arabic gum, maltodextrin, and pea protein hydrolysate. In support of food safety, contaminant assessment includes trace metals and polycyclic aromatic hydrocarbons (PAHs). In addition, contaminants regulated under EU 2023/915—including dioxins (37 congeners), dioxin-like and non-dioxin-like PCBs (DL-PCB/NDL-PCB), cadmium, mercury, and lead—will be analyzed by a certified laboratory in accordance with applicable legal limits [25].

#### 2.5.2. Comparator: Synthetic Vitamin D_3_

The comparator contains synthetic cholecalciferol (vitamin D_3_) sourced as a pharmacopoeia-compliant cholecalciferol concentrate powder (Vitamin D_3_ 100; product code 015209; CAS 67-97-0), compliant with the European Pharmacopoeia monograph “Cholecalciferol concentrate powder form”. The declared potency of the raw material is ≥100,000 IU vitamin D_3_/g (≥2500 μg/g), with defined physical specifications including particle size distribution (100% through 30 mesh; ≥90% through 50 mesh; ≤25% through 120 mesh) and loss on drying ≤ 5% (105 °C).

Supplier specifications include predefined limits for heavy metals (total ≤10 ppm; lead ≤ 0.5 ppm; arsenic ≤ 1 ppm; mercury ≤ 0.1 ppm; cadmium ≤ 0.5 ppm) and microbiological acceptability criteria (total aerobic count ≤ 1000 CFU/g; yeasts and molds ≤ 100 CFU/g; *Salmonella* negative/25 g; *Escherichia coli*, *Staphylococcus aureus*, and *Pseudomonas aeruginosa* negative/10 g; Enterobacteriaceae negative/g). The material includes food-/pharmaceutical-grade excipients, and vitamin D_3_ is sourced from wool grease (lanolin).

For the final comparator capsule, vitamin D_3_ is formulated in an oil-based vehicle using medium-chain TG (MCT) as carrier and alpha-tocopherol acetate as antioxidant within a hard gelatin capsule.

#### 2.5.3. Product Handling, Storage, and Accountability

Study products will be handled in accordance with label instructions and International Council for Harmonization Good Clinical Practice (ICH-GCP) principles. They will be stored in a dry, secure location at a controlled room temperature (≤25 °C), protected from excessive heat, moisture, and direct light, with access restricted to authorized study personnel only. Product integrity will be ensured through routine verification of packaging conditions and expiry dates prior to dispensing; any temperature excursions, damage, or other irregularities will be documented and managed according to site procedures.

Dispensing will be performed using treatment kits identified by study-specific codes to preserve allocation concealment and will be recorded in dedicated accountability logs, including participant identification, kit code, quantity dispensed, dispensing dates, and staff signature. The accountability documentation will allow full traceability of investigational product movement from receipt to final reconciliation.

At the end-of-study visit, participants will be instructed to return all empty, partially used, and unused containers. Returned products will be reconciled against dispensing records, and adherence will be assessed by capsule count, expressed as the proportion of capsules taken relative to the expected intake over the intervention period. Any discrepancies (e.g., missed returns, lost containers) will be documented. Unused study products will not be re-dispensed and will be retained and/or disposed of in accordance with applicable institutional and local regulatory requirements.

### 2.6. Randomization, Allocation Concealment, and Blinding

Randomization will be conducted in a 1:1 ratio using a computer-generated allocation sequence prepared by independent personnel not involved in participant enrolment, clinical assessment, or outcome evaluation. The randomization list will be generated using an appropriate algorithm to ensure unpredictability of assignment and will be securely stored with controlled access to maintain allocation integrity throughout the study.

Allocation concealment will be ensured through the use of pre-packaged, sequentially assigned coded treatment kits/boxes, which will be indistinguishable in appearance and labelled with study-specific identification codes only. Study products will be dispensed according to the assigned kit code, thereby preventing foreknowledge of treatment allocation at the time of enrolment and throughout follow-up.

This will be a double-blind study. Participants, investigators, study staff involved in outcome assessment, and data analysts will remain blinded to group assignment until completion of all study procedures and database lock. Unblinding will be permissible only in exceptional circumstances where knowledge of the allocated intervention is deemed essential for the clinical management of a participant and to safeguard participant safety. Any emergency unblinding will be performed via a predefined procedure, fully documented (including rationale, date, and personnel involved), and promptly reported according to applicable regulatory and institutional requirements.

### 2.7. Schedule of Assessments

**Figure 1:**
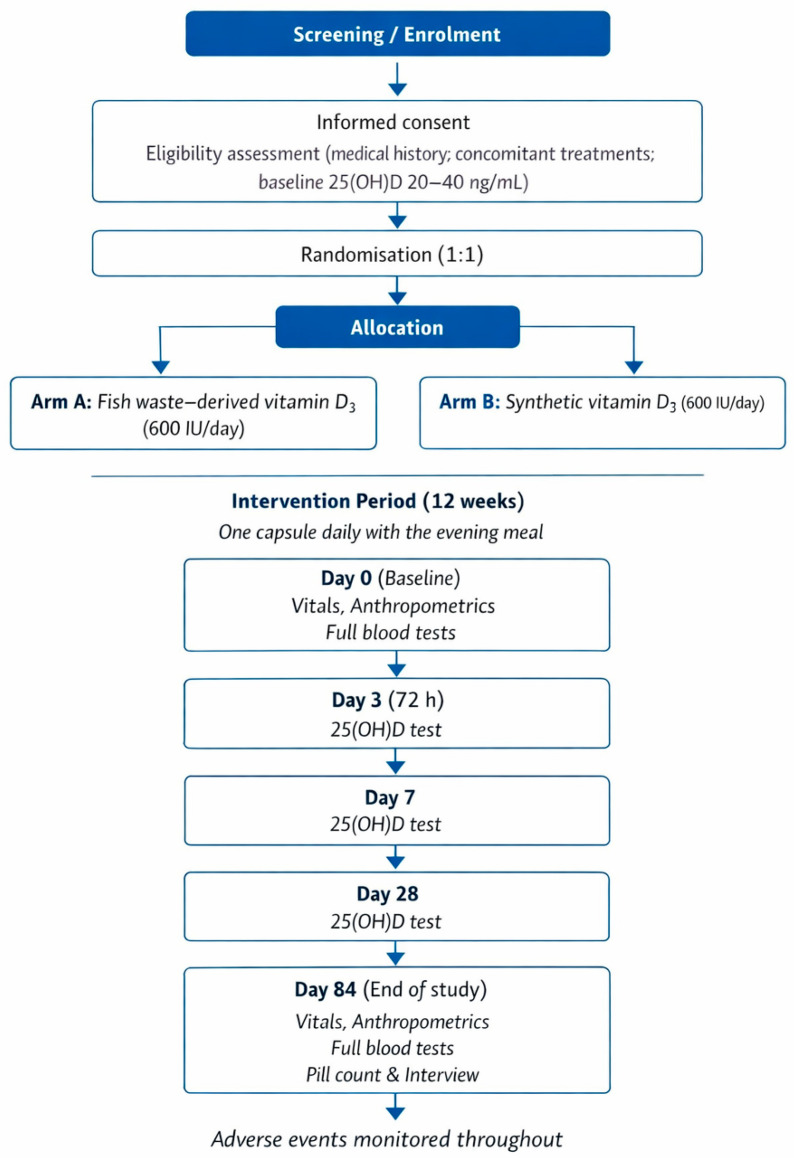
Study flow diagram of the D-FISH trial. After screening and informed consent, eligible participants are randomized 1:1 to receive fish processing by-product-derived or synthetic vitamin D_3_ (600 IU/day) for 12 weeks (one capsule daily with the evening meal). Assessments are performed at day 0, day 3 (72 h), day 7, day 28, and day 84. Adverse events are collected throughout follow-up.

The timing of enrolment, allocation, intervention administration, and study assessments is summarized in Table 2, in accordance with SPIRIT (Standard Protocol Items: Recommendations for Interventional Trials). The study flow diagram is shown in Figure 1. All parameters collected during the study will be assessed using standardized methodologies [27,28].

### 2.8. Outcomes

#### 2.8.1. Primary Endpoint

The primary endpoint is the between-group comparison of the change in plasma 25(OH)D from baseline (day 0) to the end of the intervention (week 12/day 84). This endpoint was selected to capture the biological efficacy of the fish processing by-product-derived formulation in improving vitamin D status relative to the synthetic comparator under standardized dosing conditions.

#### 2.8.2. Secondary Endpoints

Secondary endpoints are intended to characterize biological correlations of vitamin D repletion, as well as the overall tolerability and safety profile of the interventions. Changes from baseline to week 12 will be assessed for key markers of mineral metabolism, including plasma calcium, phosphorus, and parathyroid hormone (PTH). In addition, given the clinical context and the interest in potential cardiometabolic correlates, changes will be evaluated in the fasting lipid profile—total cholesterol, LDL-C, HDL-C, and TG. These measures are included as exploratory cardiometabolic correlates and to exclude clinically relevant adverse shifts, acknowledging that evidence on vitamin D-related lipid effects is mixed across cholesterol fractions and more consistent for TG. Changes will also be assessed in anthropometric measures, including body weight, waist and hip circumferences, and BMI.

Tolerability and acceptability will be captured through structured participant assessment/interview as specified in the protocol, alongside systematic collection of adverse events throughout follow-up. Safety outcomes include the incidence of adverse events and serious adverse events, withdrawals due to adverse events, and clinically relevant changes in vital signs.

#### 2.8.3. Exploratory Endpoints

To explore early response patterns, the trial will additionally examine the short-term kinetics of plasma 25(OH)D through intermediate sampling at 72 h, day 7, and day 28, thereby providing a descriptive time-course profile of vitamin D status changes under the two formulations.

All endpoints will be assessed according to the schedule reported in Table 2.

### 2.9. Adherence, Concomitant Treatments, and Protocol Deviations

Participants will be instructed to take one capsule once daily with the evening meal throughout the 12-week intervention period, in accordance with the assigned study regimen. At each contact, participants will be reminded to maintain consistent intake habits and to report any missed doses or interruptions. Adherence will be evaluated at the end-of-study visit primarily by capsule count and will be corroborated by a structured participant interview aimed at capturing dosing regularity, reasons for missed doses, and any difficulties in maintaining the prescribed schedule.

Concomitant treatments will be permitted provided they are clinically indicated and remain stable throughout the trial. Any changes in background therapy occurring during follow-up, including dose adjustments, temporary discontinuations, or initiation of new medications, will be systematically recorded. In order to minimize confounding and preserve interpretability of the intervention effect, the initiation of vitamin D-containing supplements or medications during the study period will not be allowed; participants will therefore be instructed to avoid any new over-the-counter preparations containing vitamin D.

Protocol deviations will be prospectively documented in a dedicated deviation log, including their nature, timing, and potential impact on study conduct and endpoints. Deviations will be reviewed prior to database lock by the study team in order to classify their relevance and define the per-protocol analysis set according to pre-specified criteria.

### 2.10. Safety Monitoring and Adverse Events

Adverse events (AEs) will be collected from the time of informed consent until the end-of-study visit. AEs will be captured through spontaneous participant reporting as well as through structured assessment at each study contact, including targeted questioning and review of concomitant medications and clinically relevant findings. Any sleep-related complaints potentially occurring during supplementation (e.g., insomnia or changes in perceived sleep quality) will be recorded as part of the adverse event assessment. Each AE will be documented with onset and resolution dates, severity (e.g., mild/moderate/severe), outcome, and any action taken regarding the study intervention. All events will be evaluated for seriousness and for their potential relationship to the study product based on clinical judgement and temporal plausibility. Serious adverse events (SAEs) will be managed, documented, and reported in accordance with institutional procedures and applicable regulatory requirements, with timely communication to the appropriate oversight bodies when indicated. Participants may discontinue the intervention at any time without prejudice. Criteria for withdrawal include participant request, unacceptable intolerance, intercurrent conditions requiring discontinuation, protocol deviations that may compromise safety, or safety concerns at the investigator’s discretion. Where feasible, participants who discontinue the intervention will be encouraged to complete end-of-study assessments, and all AEs will be followed until resolution, stabilization, or a clinically acceptable outcome.

### 2.11. Sample Size Determination

The planned sample size is 48 participants (24 per group). The calculation is based on a non-inferiority framework for the primary endpoint, assuming a standard deviation of 5.5 ng/mL for the change in 25(OH)D, a non-inferiority margin of 5 ng/mL, 90% power, and an allowance for approximately 10% drop-out and exclusions due to non-compliance. The non-inferiority margin (5 ng/mL, ~12.5 nmol/L) was prespecified to represent a conservative and clinically acceptable difference in biochemical response between formulations at nutritional doses while preserving feasibility for this proof-of-concept trial. Although no universally accepted minimal clinically important difference for 25(OH)D change exists, the margin was selected a priori to avoid differences likely to be regarded as meaningful in routine biochemical monitoring.

### 2.12. Statistical Analysis Plan

Analyses will be conducted in predefined analysis populations: a full analysis set (FAS; intention-to-treat principle), a per-protocol (PP) set excluding major protocol deviations, and a safety set including all participants who receive at least one dose of study treatment.

For the primary endpoint, non-inferiority of fish-derived vitamin D_3_ versus synthetic vitamin D_3_ will be concluded if the lower bound of the confidence interval for the between-group difference (fish-derived minus synthetic) in the change in 25(OH)D at week 12 is above −5 ng/mL. A repeated-measures ANCOVA model will be used, with treatment group and visit as factors and baseline 25(OH)D as a covariate. The primary analysis will be performed in the FAS and repeated in the PP set as a sensitivity analysis.

Secondary continuous outcomes will be analyzed using ANCOVA models with treatment group as factor and baseline value as covariate (or appropriate non-parametric alternatives if model assumptions are not met). Categorical outcomes (e.g., AE incidence) will be summarized as counts and percentages and compared using appropriate tests where relevant. As an exploratory sensitivity analysis, the month/season of blood sampling may be included as a covariate to assess the robustness of the primary comparison. No interim analysis is planned.

### 2.13. Data Management and Confidentiality

Study data will be recorded in a study-specific electronic case report form (eCRF) hosted on REDCap-Unibo, with role-based access control, individual authentication, and full audit trails to ensure traceability of data entry and modifications. Data handling will follow the ICH-GCP principles and applicable data protection requirements [29,30]. Participants will be identified in the database by a unique study code, and any direct identifiers will be kept separate from research data whenever applicable, with access restricted to authorized study personnel. Data quality and integrity will be supported by predefined validation rules and structured data fields, together with quality control procedures including range and logic checks, query generation and resolution, and periodic review of completeness and internal consistency. Data exports for analysis will be performed in a controlled manner, limiting access to pseudonymized datasets. Study records will be stored securely and retained/archived according to institutional policies and applicable regulations, ensuring confidentiality, controlled access, and data availability for monitoring and verification activities where required.

### 2.14. Ethics and Dissemination

The trial will be conducted in accordance with the principles of the Declaration of Helsinki [31] and ICH-GCP [29]. The study protocol has been submitted for review to the Comitato Etico Area Vasta Emilia Centro (AVEC), and all required institutional authorizations will be obtained prior to participant recruitment. Written informed consent will be obtained before any study-related procedure.

Study findings will be disseminated through peer-reviewed publication and presentation at scientific conferences, irrespective of the study outcomes. The trial has been registered on ClinicalTrials.gov (identifier NCT07127796) [27].

### 2.15. AI Use Acknowledgement

The graphical abstract of this paper was created using Google Collaboratory (Google Notebook). ChatGPT 5.2 was used exclusively for language editing purposes. The same will be done with the final report of the study.

## 3. Discussion

D-FISH addresses a timely question with combined clinical and sustainability relevance: whether a vitamin D_3_ supplement derived from fish processing by-products (valorization of materials otherwise discarded) can achieve improvements in plasma 25(OH)D that are not clinically meaningfully lower than those obtained with a conventional synthetic vitamin D_3_ comparator when administered at an equivalent nutritional dose. Importantly, D-FISH is designed to test biochemical non-inferiority in 25(OH)D response and is not powered to evaluate clinical outcomes; therefore, no conclusions regarding clinical benefit can be drawn from this trial. If non-inferiority is demonstrated, the trial will provide an evidence-based rationale for considering circular-economy approaches to vitamin D nutraceutical production while preserving expected biological efficacy—a paradigm that is increasingly relevant as nutraceutical development expands beyond efficacy alone to include broader value dimensions.

Nevertheless, the primary endpoint is clinically meaningful. In routine practice, 25(OH)D is the accepted biomarker used to diagnose suboptimal vitamin D status and to guide supplementation strategies, monitoring, and dose adjustment. In this context, demonstrating non-inferior biochemical repletion represents a necessary translational prerequisite before considering broader implementation or investing in larger outcome-driven trials. Moreover, direct randomized, double-blind head-to-head comparisons between by-product-derived and synthetic vitamin D_3_ formulations at nutritional doses remain scarce, despite growing interest in sustainable sourcing and production pathways.

The choice of a non-inferiority framework is particularly appropriate in this setting, where the investigational product is not primarily expected to outperform the standard comparator but rather to provide comparable biological benefit while potentially offering advantages in sustainability, sourcing, and production. Non-inferiority trials have become a major tool in modern clinical research when an effective standard exists and placebo-controlled comparisons would be unethical or uninformative; their original rationale was to identify “good substitutes” that preserve efficacy while improving other attributes such as convenience, safety, or cost [32]. At the same time, non-inferiority designs require careful methodological execution and interpretation, given their complex assumptions and the potential for design or reporting features to bias inference toward concluding non-inferiority [33,34]. Evidence from the high-impact cardiovascular literature highlights that non-inferiority trials have historically varied widely in margin selection and frequently presented methodological or reporting limitations—particularly when results were not consistently reported across intention-to-treat and per-protocol/as-treated analyses [35,36]. Against this backdrop, D-FISH incorporates key safeguards to strengthen interpretability, including a prespecified non-inferiority margin and confirmation of the primary analysis in both full analysis and per-protocol populations. The selected margin was defined a priori to represent a conservative difference in 25(OH)D change that would be unlikely to be regarded as clinically meaningful in routine biochemical monitoring, thereby supporting a more robust inference even in the presence of potential deviations that could dilute between-group contrasts [34,35].

Several additional design features further support interpretability. The randomized, double-blind, parallel-group structure reduces selection and ascertainment biases and supports unbiased outcome assessment. The primary endpoint—change in 25(OH)D from baseline to week 12—directly captures the intended biological effect of supplementation and aligns with real-world practice, where 25(OH)D remains the standard biomarker used to evaluate vitamin D status. Importantly, the investigational formulation is supported by a structured upstream workflow, including controlled raw material handling, supercritical CO_2_ extraction, analytical characterization and standardization, and microencapsulation, together with a dedicated safety testing program addressing relevant contaminants. From a translational standpoint, this quality-by-design and traceability approach is a non-trivial component of sustainability-oriented nutraceutical development, as it helps ensure that environmental advantages do not come at the expense of reproducibility, standardization, or safety.

The intermediate sampling time points (72 h, day 7, and day 28) represent an additional asset. Although the study is not designed for mechanistic modelling of absorption, distribution, or compartmental kinetics, these early measurements provide a pragmatic description of short-term 25(OH)D response under daily administration. This may help contextualize potential differences in response trajectories between formulations that differ in source matrix and processing and may generate hypotheses for future research aimed at clarifying determinants of early response patterns under nutritional-dose supplementation.

Limitations should also be acknowledged. The trial is single-center and is powered for a biochemical non-inferiority endpoint rather than clinical outcomes; thus, no conclusions regarding patient-relevant benefit can be drawn. The 12-week duration—while appropriate for capturing meaningful changes in 25(OH)D at nutritional doses—does not inform longer-term maintenance, seasonal variability, or downstream endpoints. Moreover, eligibility criteria restrict enrolment to adults aged 18–60 years with baseline 25(OH)D 20–40 ng/mL, which supports homogeneity but limits generalizability to older adults, individuals with more severe deficiency, or those with major comorbidities. In addition, the trial does not formally control for or adjust analyses by individual sunlight exposure, seasonal variability, dietary vitamin D intake, or body composition, which may contribute to between-individual variability in 25(OH)D response. As a consequence, seasonal fluctuations and day-to-day variability in UVB exposure may influence individual trajectories in 25(OH)D despite randomization. Finally, since the protocol includes a fixed evening-meal administration schedule, the trial is not designed to evaluate whether different circadian timing strategies may influence tolerability or biochemical response. Moreover, although tolerability and safety are systematically assessed, the sample size and follow-up are insufficient to draw definitive conclusions regarding rare or longer-term adverse events, and safety findings should be interpreted as short-term tolerability signals.

In conclusion, D-FISH is a randomized, double-blind non-inferiority trial designed to evaluate whether a fish processing by-product-derived vitamin D_3_ formulation produces a change in 25(OH)D that is not clinically meaningfully lower than a synthetic comparator at the same nutritional dose. The study will inform short-term biochemical efficacy and tolerability, but it is not powered to assess clinical outcomes or establish interchangeability. Larger multicenter, longer-term, outcome-driven trials will be required before any broad clinical adoption can be considered.

## Figures and Tables

**Table 1:** Eligibility criteria.

Section	Criteria
Inclusion criteria	(1) Men or women aged 18–60 years (2) Plasma 25(OH)D between 20 and 40 ng/mL (3) Ability to understand study procedures and comply with trial requirements (4) Written informed consent provided prior to any study-related procedure
Exclusion criteria	(1) Current intake of vitamin D-containing supplements or medications (2) Osteopenia/osteoporosis, rheumatologic diseases, or history of non-traumatic fractures (3) Known hyperparathyroidism, hypercalcemia, or severe renal failure (eGFR < 30 mL/min/1.73 m^2^) (4) Known fish allergy/intolerance or allergy to fish-derived products (5) Preventive drug treatments (e.g., lipid-lowering or antihypertensive agents) not stable in type and dose for ≥3 months (6) Pregnancy or lactation (7) Any condition likely to compromise adherence to the protocol (including clinically relevant malabsorption syndromes)

25(OH)D, 25-hydroxyvitamin D; eGFR, estimated glomerular filtration rate.

**Table 2:** SPIRIT (Standard Protocol Items: Recommendations for Interventional Trials) schedule of enrolment, interventions, and assessments for the D-FISH trial.

Procedures/Assessments	Enrolment/ Baseline (Day 0)	72 h (±1 Day)	Day 7 (±1 Day)	Day 28 (±3 Days)	End of Study (Day 84 ± 5 Days)
Informed consent	X				
Eligibility assessment	X				
Randomization (1:1)	X				
Dispense study product	X				
Study product intake (daily, 600 IU/day)	X	X	X	X	X
Concomitant therapies review	X				X
Vital signs	X				X
Anthropometrics (weight, height, waist, hip; BMI)	X ^†^				X ^‡^
Adverse events assessment	X	X ^§^	X ^§^	X ^§^	X
Tolerability/acceptability interview					X
Pill count/accountability					X
Blood sampling: 25(OH)D	X	X	X	X	X
Blood sampling: calcium, phosphorus, PTH	X				X
Blood sampling: lipid profile (TC, LDL-C, HDL-C, TG)	X				X

^†^ baseline anthropometrics include also height for BMI calculation; ^‡^ end-of-study anthropometrics include weight and circumferences, BMI will be recalculated; ^§^ brief AE/tolerability check may be performed during intermediate sampling, according to site logistics. Abbreviations: 25(OH)D, 25-hydroxyvitamin D; AE, adverse event; BMI, body mass index; HDL-C, high-density lipoprotein cholesterol; LDL-C, low-density lipoprotein cholesterol; PTH, parathyroid hormone; TC, total cholesterol; TG, triglycerides.

## Data Availability

No new data were created or analyzed in this study. Data sharing is not applicable to this article.
